# Using Link Disconnection Entropy Disorder to Detect Fast Moving Nodes in MANETs

**DOI:** 10.1371/journal.pone.0155820

**Published:** 2016-05-24

**Authors:** Carlos F. Alvarez, Luis E. Palafox, Leocundo Aguilar, Mauricio A. Sanchez, Luis G. Martinez

**Affiliations:** Faculty of Chemical Sciences and Engineering, Autonomous University of Baja California, Baja California, Tijuana, Mexico; Nankai University, CHINA

## Abstract

Mobile ad-hoc networks (MANETs) are dynamic by nature; this dynamism comes from node mobility, traffic congestion, and other transmission conditions. Metrics to evaluate the effects of those conditions shine a light on node’s behavior in an ad-hoc network, helping to identify the node or nodes with better conditions of connection. In this paper, we propose a relative index to evaluate a single node reliability, based on the link disconnection entropy disorder using neighboring nodes as reference. Link disconnection entropy disorder is best used to identify fast moving nodes or nodes with unstable communications, this without the need of specialized sensors such as GPS. Several scenarios were studied to verify the index, measuring the effects of Speed and traffic density on the link disconnection entropy disorder. Packet delivery ratio is associated to the metric detecting a strong relationship, enabling the use of the link disconnection entropy disorder to evaluate the stability of a node to communicate with other nodes. To expand the utilization of the link entropy disorder, we identified nodes with higher speeds in network simulations just by using the link entropy disorder.

## Introduction

A Mobile Ad-hoc Network (MANET) is a wireless network made of multiple mobile devices called nodes; with no need for a fixed infrastructure; capable of self-organization and easy to deploy in an instant [1]. All devices are free to move, having total independence with the rest of devices; forming a dynamic topology of moving devices.

All participating nodes in a MANET are resource independent. Each node has the resources needed to operate without the need of others.

Node independence can provide the flexibility to disperse the nodes without a particular pattern on a working area. The only restriction is to have some other participants at transmitting range, needed for forwarding packets to out of range nodes.

The dynamic topology of the MANET is ideal for some military and commercial applications such as scouting or swarming, disseminating communication or sensory detection capabilities in a localized area.

The connection effectiveness between nodes influences the MANETs stability to communicate. The node‘s mobility disturbs the efficiency of delivering packets. A MANET with little movement in nodes is communication stable; when nodes move rapidly, the network needs to adapt to maintain stability.

A fast moving device is continually creating new node connections when in the transmission range with a neighbor, but when it is needed to use the link, the fast node is not in the same area. This rogue device makes the communication difficult for nearby nodes presenting a short-lived connection.

Identifying and measuring rogue nodes could be valuable. By identifying a fast rogue node, we can take this node out of the routing arrangement or stop the participation of this device in the network. Another usage is detecting nodes with “out of the ordinary” behavior like malfunctioning sensor nodes.

When simulating MANETs, two networks cannot be easily compared, due to the complex interaction between system parameters. Any difference in working area size could leave nodes too apart to create a reliable link, or too close putting more nodes in range to connect. Considering two networks of different size and node speeds, we can compare them by observing traits of the system, such as when they have the same connection interactions, regardless of difference in the parameters; this comparison can be done with a specific trait and handling each system as a graph and quantitatively investigate them as network-based systems [2].

Multiple metrics and indexes exist to evaluate the reliability and the impact of MANET’s mobility. Some metrics and indexes use the location, speed, or distance to assess mobility [3–7]. For this, extra CPU and power consumption is needed to use of additional sensors. Others, use specific marks in communications such as Time to live packets or packet return time; in some cases, using a great deal of the bandwidth in the control packages.

This paper expands the description and implements previous work by the authors[8]. The proposed index use the entropy of all the link’s disconnections to nearby neighbors within a timeframe. Without the need for additional sensors and little use of resources built-in in most basic devices for this type of networks.

Applying the index in a single node, it will measure the node stability to communicate with others owed mostly to mobility.

A high level of Link Disconnection Entropy Disorder (LDED) points to an unstable group of neighbors and the connections to them. Bigger LDED is an effect of adding connections to new neighbors and the breaking of links to previously associated nodes, as in a node moving rapidly through the network area.

When the LDED is small, the presence and communications are stable as the nodes do not change positions for longer periods of time. If the nodes in the network are not moving, there is no change in neighbors or connections.

To evaluate the network Link Disconnection Entropy Disorder, the index uses the individual LDED evaluated by each node in the MANET, creating a reference point for comparison of two systems with different parameters. If a system with a large working area has the same LDED such as a smaller one with fewer nodes, they can be considering similar systems.

In section “Mobile Ad-hoc NETworks” we define the use and importance of this type of system; detailing the frequently used metrics is in section “Metrics in MANETs”. The basis for the metric are in “Entropy and Disorder”; in “Link Disconnection Entropy Disorder” is the workings of the metrics to use entropy disorder to measure node stability. The technical details of simulations are in “Simulation basics” section used in “Results”, finishing with a conclusion.

## Materials and Methods

### Mobile Ad-hoc NETworks

MANETs provide the possibilities for applications where a fixed infrastructure is not available, by forming a dynamic and extemporaneous one; usually for special or customized application.

In the battlefield, autonomous agents could be able to assist in communications and data gathering for intelligence, like surveillance, damage assessment, and other tactical needs.

For civilian life, some proposals are published; one of this proposals is a dispatch system for taxis [9] while another application is a complete framework for applications to monitor the neighborhood [10]. The vehicular industry is one to benefit using in-vehicle network applications as in vehicle-to-vehicle communications.

In this type of network, all devices have basic resources to do sensing as a standalone node, but when the time is to communicate, they need a communications infrastructure to send or receive data. All participating nodes form a temporary and dynamic communications support by using a nearby neighbor as a forward point to other nodes.

**Fig 1:**
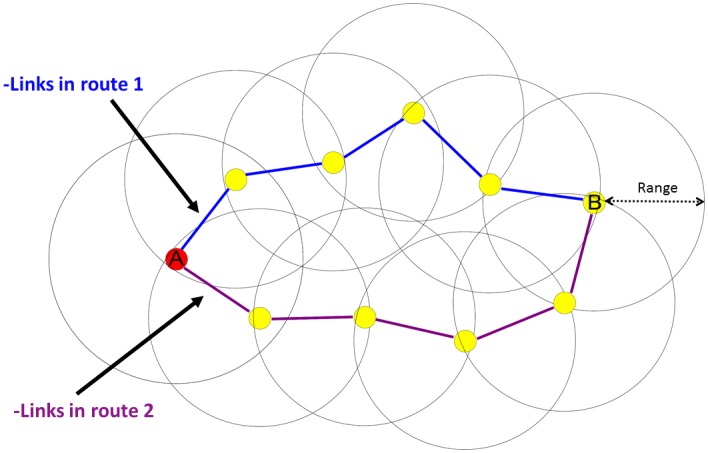
Basic MANET with multiple hops. Node "A" requires to transmit to node "B", two route options are available.

Shown in Fig 1, are the two possible paths to communicate node “A” to node “B”. Node “A” uses in-range neighbors to form a potential forwarding path of nodes to node “B”.

When a transitional node fails, the network reacts searching for other nodes to help the package reach the destination. If the node participating in forwarding is turned off or goes missing, the last node in the path looks for a new suitable node to continue passing the package.

A routing protocol is needed to coordinate all nodes in a distributed manner, considering all nodes participate in forwarding packets; leaving each device a part of the responsibility of selecting the next node to communicate. Various routing protocols exists [11–15]. The routing protocol is the responsible for coordinating routes and error messages using a particular logic to select the nodes participating in a path to nodes out of range.

One main characteristic of MANETs is node mobility. Nodes constantly move at different rates, changing the possible paths to use or breaking the existing ones. The routing protocol needs to adapt to the changing conditions and react when needed.

The selection of an individual path to other nodes is made by choosing the path with the best metric defined by the routing protocol. This metric varies depending on the routing protocol selected. A metric could evaluate each node, the complete list of nodes in a path, or all the links involved in the route.

### Metrics in MANETs

A metric or index needs to model the quintessential characteristics of each node in the system. A good metric could furthermore describe the behavior of the network and the elements enclosed; for that it could be used as a similarity measure or to describe a node.

Graph similarity measures[16,17] are used to compare systems where no comparison point can be easily obtained by parameters alone; some measures are based on topological characteristics [18,19], others measures are based in entropy [20–22]. Emmert-Streib et al. [23] did a comprehensive survey of methods for comparative graph analysis.

When comparing two MANETs, any operational area size difference denotes different density of nodes affecting the neighbors in range and channel saturation due to crowding, making it difficult to compare due to dissimilar interactions. Only two networks with the same similarity measure can be considered analogous, even with different parameters. Graph entropy measures can be used as a similarity measure.

Understanding node’s mobility and dependability through metrics and indexes prepare us to adapt or just recognize how the system is performing. Evaluating provides feedback from the network by quantifying the effect of node consistency [24].

The use of Creative measurements is needed to assess trustworthiness in nodes with fewer resources. Power consumption could be minimized by using data already known by the node, with less CPU utilization and lower resource use. These creative metrics describe link or path stability or link behavior [25–27].

In this type of wireless network, the nodes are free to move independently of each other. The MANET topology changes as the mobile devices travel through the working area, disturbing data rates and links between nodes, disappearing established links of communication [25].

Ad-hoc metrics derive from the behavior of links when affected by movement. As the node moves, links to near neighbors change performance. When a connection is completely stable, always present, the communication is at optimal.

When a connection keeps breaking for half the time, then half of the packages passing through are not delivered or are retransmitted, degrading the link. ETX [26,28] use the relationship of forward and reverse delivery ratio of the connection; link duration metric (LD) [29] uses the term of each existing link between nodes. Some others evaluate the rate of change of one or more characteristics [3].

A measure that can tell the effect of movement on the node’s links could help to understand and react the network’s dynamics.

If a routing protocol uses a metric or index, it could support the selection of the most stable route from a group of paths. The metric or index describe the dynamics of each node in the network.

A practical mobility measure needs to cover some real world characteristics [30]:

- Must apply to real nodes
- Has to be computable in a distributed environment
- Adapt to measure performance locally
- Numerically quantify the quality of a link
- Evaluated in real time

The index proposed here could be applied to evaluate a single node, an established route, or the nodes participating in a path. The resulting LDED when using all links in a path will assess the stability of the complete path. To determine how stable is the group of nodes forwarding as a team, the LDED of each node involved in a path is used.

### Entropy and disorder

Entropy is a versatile metric [31] used in different fields. This metric is used and adapted to chemistry, biology and medical research [32,33], animal and human behavior [34–36].

In computer science, it is used in analysis of complex systems and information [37–40], Image analysis [41], and computer networks [3,42,43].

As stated by Emmert-Streib [23] “Graph entropy measures are multivariate function capturing information beyond distances” making graph entropy measures a good fit to evaluate MANETs. Graph entropy measures have been classified and contextualized by Mowshowitz et al. [44].

Entropy-based metrics in ad-hoc networks uses a rate of change or behavior to create a measure; usually to entropy, order, or disorder. Entropy presents the uncertainty in a system [4,6,42]. The majority of Entropy measures in Ad-hoc Networks rate the changes in location or link characteristics such as breakage. When a measurement is significant on entropy means high rates of change [45].

Disorder [46,47] is a normalized representation of entropy. The definition of Disorder is [45,48]

$$\Delta \;=\;\frac{S}{S_{max}}$$ (1)

Where S is the Boltzmann-Gibbs-Shannon entropy[49], given by:

$$S=\;-k\;\sum_{i=1}^{N}\left(p_{i})\;{\text{log}}_{2}(p_{i}\right)$$ (2)

Using *p*_*i*_ as the probability of state i of the N states in the system, the Boltzmann constant is represented by k.

*S*_*max*_ Denotes the maximum entropy of a system with equiprobable distribution of *p*_*i*_ [46,47]

$$S_{max}=\left(k\right)\;{\text{log}}_{2}(N)$$ (3)

N being the number of possible states in the system and k the Boltzmann constant. The *p*_*i*_ value for each possible state is

$$p_{i}=\;\frac{1}{N}$$ (4)

### Link disconnection entropy disorder

Specialized sensors are needed to measure physical movement (Speed, Acceleration, position), using significant power resource (Battery). It is possible to observe the effect of movement on the connections to determine the stability of the node due to mobility.

Node connections can be monitored by periodically sending a simple Hello package. The hello package is overheard only by neighbor nodes in transmission range; registering a possible connection state; when it is missing at a designated time, it is expected to be lost by being out of range.

The Hello beacon package could be small, it does not need much information. The objective of this package is to inform near neighbors of the node presence, with it, the possibility of a connection. The periodicity of the beacon could be key to account for lost packets in an observed time T. Each participating node notifies his presence to near neighbors and a possible connection.

**Fig 2:**
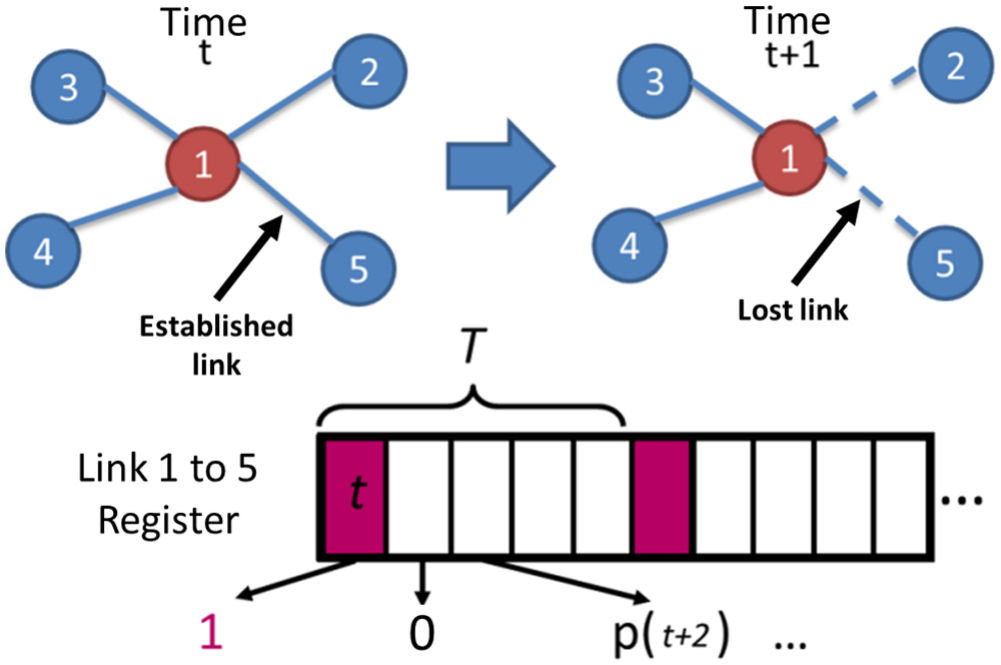
Node observation time window. Node 1 in time t+1 drops connections made with node 2 and five at time t; registering success or failure in an array with a sliding time frame.

In Fig 2 a beacon is received, marked as 1 in time t is a successful link. Links existing at time t are broken in time t+1; the expected Hello package did not arrive at expected time p(t+1). A register of the received or lost beacon is used to evaluate the entropy micro-states during time window T.

The time window T is a sliding window where each beacon data is collected and kept for a designated time, discarding entries outside this period. Using a 4 seconds time window, entries older than this are discarded.

When evaluating LDED, we are using the count of the missing packages, therefore if we expected ten beacons but only received 4, we expect a six package loss in that time frame.

**Fig 3:**
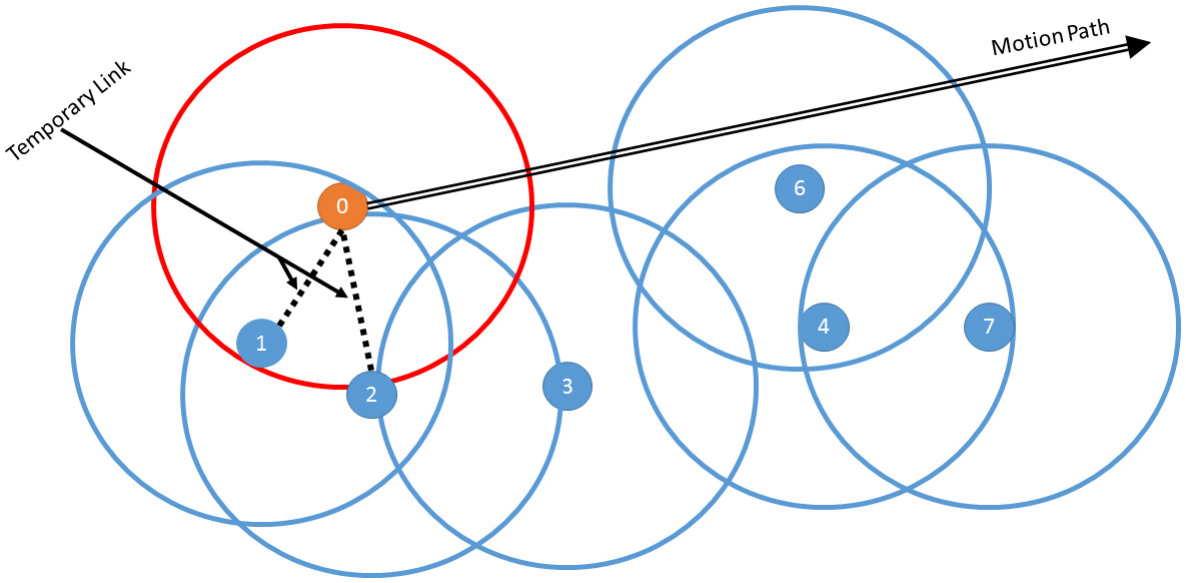
Node is moving through network. Node is moving along the network, creating link breakage, and new node connections.

When a node moves inside the network area, link breakage and newly made connections take place. Fig 3 shows moving node 0 with a fixed direction and speed. When the node passes in range of a neighbor, a new link register is created for each newly connected node.

The link register array exists while not empty with success connections to the node in the time frame. The link record is eliminated when no entry is received in the period. All link records to neighbors with at least one entry are used to measure the nodes LDED.

If the connection is sustained throughout the time frame, the link disconnection disorder is small; denoting little or no change is made to the link. When the connection is lost and regained several times in the time window, link disconnection entropy disorder will elevate.

The total of possible connections and disconnections are used to evaluate the LDED of a node with neighbors. A node is considered stable when the possibility of connection is present during the time window. On the other hand when most of the time the likelihood of connection is small, denotes an unstable node to communicate with neighbors, meaning an uncertainty for future connection.

One notable characteristic of a moving node is the constant creation of links to newly seen neighbors. New entries for links from recently encountered neighbors are added to the possible future connections, changing the node’s entropy elevating the LDED with each new node.

If a neighbor is without a successful connection during the time window, that neighbor, and all the possibilities for connections are not included for evaluation.

Using N as the number of expected connections by a node during a time window in eq 3, the maximum entropy is calculated. Eq 2 evaluates the node’s disconnection entropy using the missing links.

The proposed Link Disconnection Entropy Disorder index has all the characteristics of a practical metric. Easy to implement in real nodes due to simple calculations, it does not require a high power processor; each node evaluates own index, distributing the processing to each node.

### Simulation Basics

Due to the complexity in randomly controlling the speed and direction for a group of wireless communication devices, a wireless network simulator is used. A testbed of reasonable size is complex and costly to deploy; in a testbed it is difficult to change protocols in a controlled and reproducible manner [50].

The framework for the simulation was Omnet++ 4.6b1[51] discrete network simulator. The libraries used to mimic wireless devices in this framework are designed to account for aspects of communications.

The physical characteristics of nodes are considered by the models, including radio communications, radio distribution and MAC protocols; therefore, the repercussions of using a simulation environment over a testbed are minimal.

OMNET++ Performance evaluation of ad-hoc networks have being discussed by Sommer et al.[52]; Bredel et al.[53] compares IEEE 802.11g measurements to OMNET++ simulations; Colesanti et al. [54] published a comparison of Simulation and testbeds.

With the objective of validating LDED, several network simulations were realized. In sets of four and seven runs, changing the random seed at each run, obtaining reproducible results. Simulation results are shown in “Results” section, OMNET++ configurations are set in this section.

RandomWaypoint mobility model [55,56] moves each node independently inside the working area. Each node selects a target destination and moves with a specified speed, when the destination is reached, a new random target goal is set, repeating the process throughout the simulation time. No waiting time was used.

**Fig 4:**
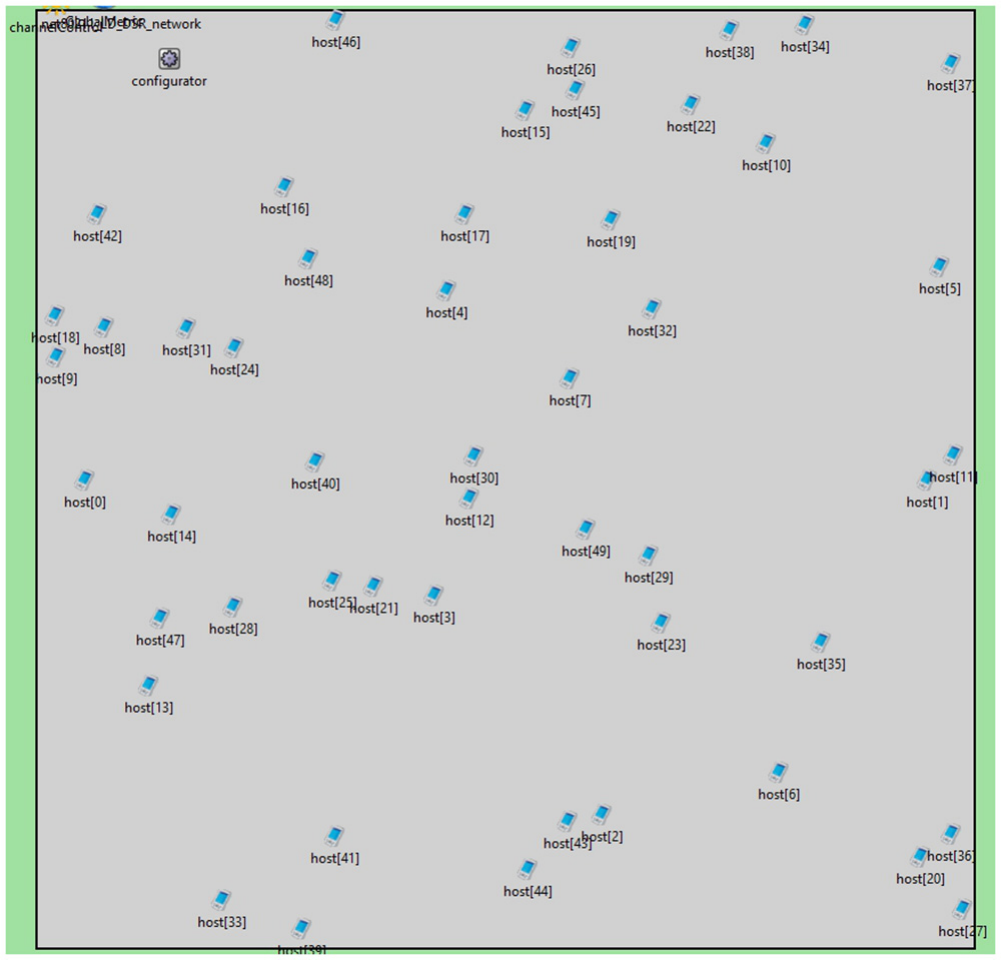
Nodes randomly distributed in 1km by 1km working area. Nodes are free to move randomly inside.

Each simulation comprised 50 mobile devices with 802.11g capabilities; each node is independent of each other and can move freely; nodes are free to transmit if needed using 802.11g (wifi) as shown in Fig 4.

To Stress the network, when in need of traffic the G.728 (16 kbps) standard is used. With a payload of 60Bytes, containing 30 seconds of audio; sending 33.3 packets per second. This traffic is working with a constant bit rate of audio for the duration of the simulation.

The parameters to evaluate LDED in each node are:

- Each node has a time window of 4 seconds.
- A single beacon every 0.5 seconds.
- Refreshing the metric every 2 seconds.

To identify the impact of speed, simulations with different speed scenarios. Each simulation run time is 600 seconds with a selected speed i.e. 0.2, 0.5, 0.7, from 2 to 10, 12, 14, 16, 18, 20, 22, 24, 26, 28 meter per second.

Three experiments were planned to evaluate LDED stability. Seeking consistency in the measurements, especially to variations in the node’s mobility; being mobility the predominant factor for communication instability in a node.

To focus on the behavior of LDED to the saturation of the communication channel and the mobility of the nodes, the first experiment was implemented. In this experiment, simulations are performed with speed and channel saturation as the variables. To saturate the communication channel four levels of saturation were used i.e. 0, 1, 5 and seven nodes transmitting to a constant bit rate of 16kbps (G.728).

The second experiment is an extension of the first. With the channel saturation and speed of the nodes as variables, analyzing the relationship between packet loss and LDED; Observing mobility and channel saturation effect size within the LDED.

The aim of the third experiment is to identify nodes with link disconnection entropy disorder alone; Comparing the node’s LDED with the LDED of reference nodes. Each simulation group uses a different speed for reference nodes. Being LDED tightly coupled with neighbor’s speed, since LDED uses the links with neighboring nodes and these nodes also move independently at own speed.

Each identifiable node moves with a fixed speed, i.e. Node A is static (0 m/s), Node B uses 2 m/s, Node C is 4 m/s, and Node D has a speed of 12 m/s. The rest of the nodes receive the reference speed for that simulation run.

To assess the reliability of the LDED, an Intraclass Correlation Coefficient (ICC) was used for each simulation group; the expected value is near 1.0, meaning a consistent value through all simulation runs.

## Results

In this section, the Link Disconnection Entropy Disorder measurements are shown as the outcome of the controlled simulations, identifying the reliability of LDED.

**Fig 5:**
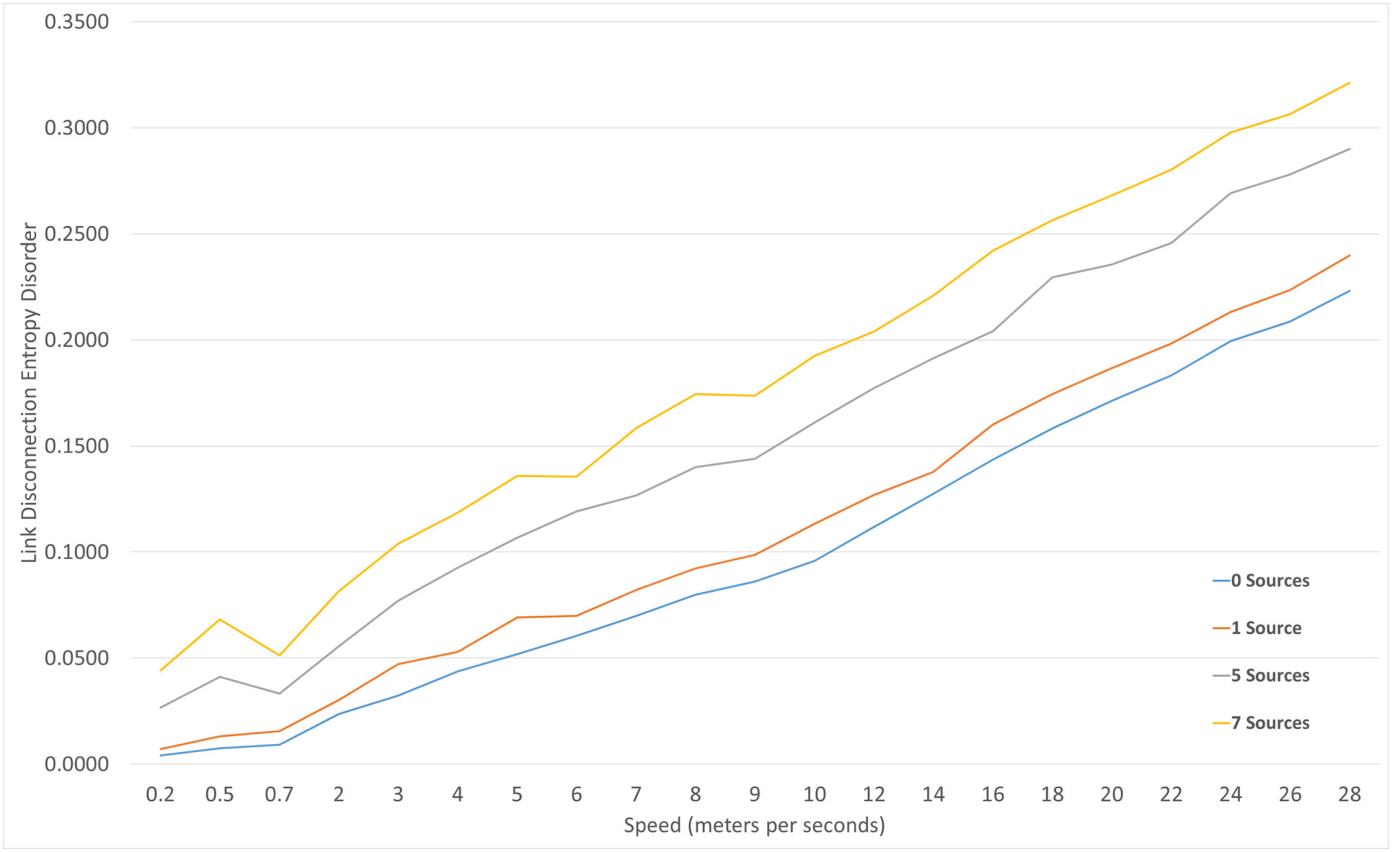
Link disconnection entropy disorder reaction to source saturation and node mobility.

As the neighboring nodes move along the working area, possible links are made and broken; this is key in evaluating a single node’s LDED. When a node is passing by new neighbors constantly, the LDED value is high. Fig 5 reveal the increment in Link Disconnection Entropy Disorder while Traffic and speed increases; Since part of the measurements involve sending a package to identify neighbors, four different channel saturation intensities were implemented to observed the influence while detecting neighbors; as the Fig 5 indicates, it is partially affected.

There is a strong relationship between the spatial mobility of a node and the link disconnection with neighbors as shown in Fig 5; if a network is high in LDED value, it is expected to have a low packet delivery ratio.

**Fig 6:**
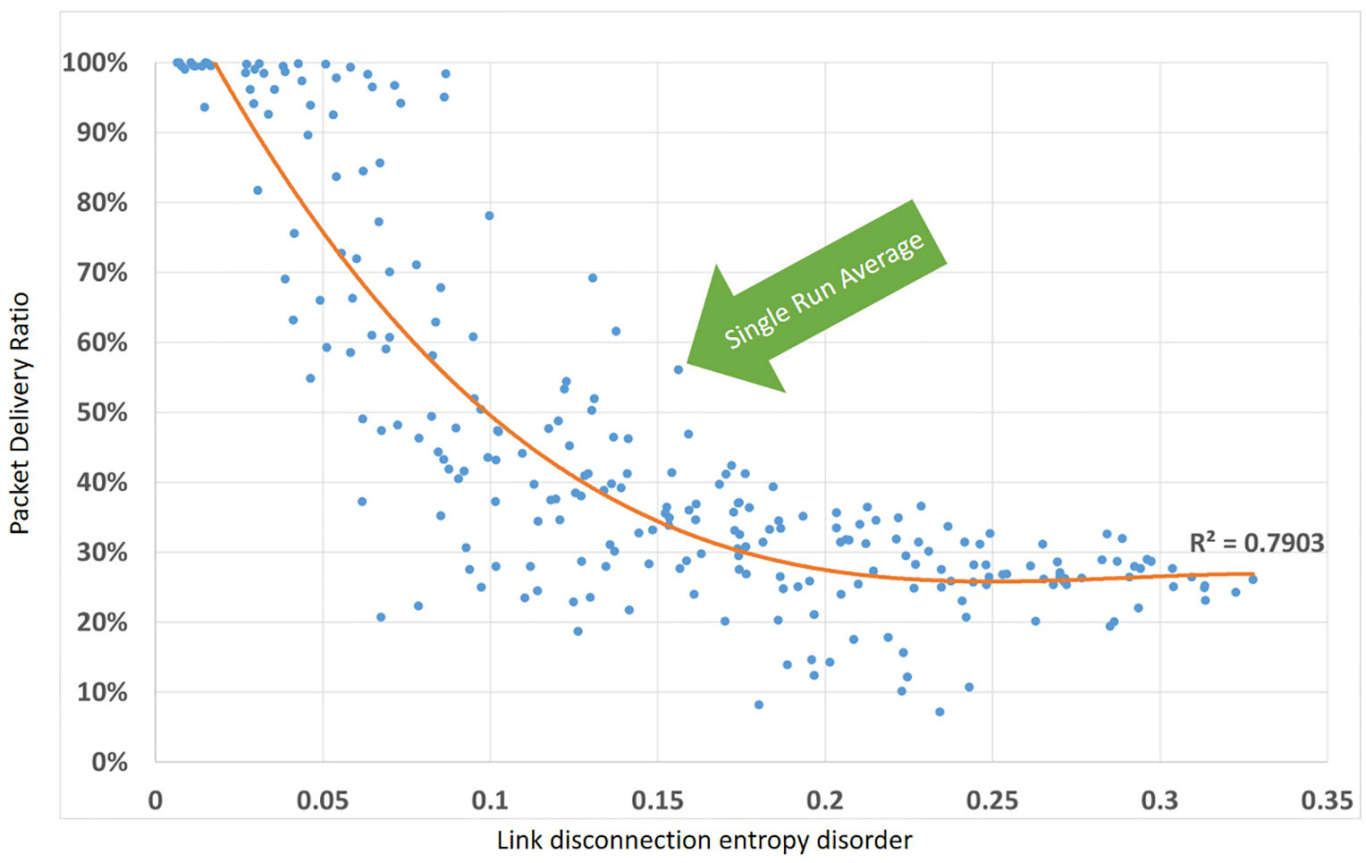
packet delivery ratio and Link Entropy Disorder.

Fig 6 illustrate the relationship between the packet delivery ratio (PDR) and Link Disconnection Entropy Disorder; the PDR gets lower as the LDED goes higher. A logarithmic trend shows a LDED relationship with PDR.

A two-way ANOVA evaluates the speed and traffic saturation’s effect size on Link Entropy Disconnection Disorder; Table 1 contains the two-way ANOVA analysis with effect size for traffic and speed as ETAsqr.

**Table 1:** Link disconnection entropy disorder two-way ANOVA result with Traffic and speed as variables.

*Source of Variation*	*SS*	*df*	*MS*	*F*	*P-value*	*F crit*	*ETAsqr*
Traffic	0.27801	3	0.0927	1630.3	6E-124	2.6584	0.1557
Speed	1.48286	20	0.0741	1304.4	5E-173	1.6334	0.8306
Interaction	0.01491	60	0.0002	4.3715	3E-14	1.3981	0.0084
Within	0.00955	168	6E-05				
Total	1.78534	251					

The ETAsqr attribute 83.06% of LDED to changes in Speed, 15.57% is to traffic saturation and less than 1% to the interaction between the two.

LDED helps to identify nodes that are moving at higher speeds than neighbors. Table 2 uses experiment three with 47 reference nodes at 0 meters per second, nodes B, C, and D use a different speed to identify them in four simulation runs. Nodes with faster speeds than reference would have a higher LDED. The difference in LDED is represented in by column “% Ref”.

**Table 2:** Node LDED comparison to reference nodes at 0mps base speed.

Node	Speed	Run 1	Run 2	Run 3	Run 4	Average	% Ref
A	0mps	0.01578	0.01506	0.01062	0.01685	0.014578	-10%
B	2mps	0.05134	0.05279	0.04475	0.05115	0.050008	209%
C	4mps	0.08387	0.09536	0.07810	0.08253	0.084964	426%
D	12mps	0.18970	0.19170	0.18332	0.20141	0.191533	1085%
R. Nodes	0mps	0.01667	0.01533	0.01543	0.01723	0.016164	0%
ICC	0.995097						

The LDED average of Node A is 10% below the reference node’s average LDED; not substantial in contrast to the 1,085% for the Node D, with faster speed than the reference nodes. Nodes B and C reflect a higher difference, identifying the fast moving nodes in relation to network’s speed average.

For this simulation group, the reliability for LDED is 0.995, given by the intraclass correlation coefficient.

Tables 3 and 4 are simulation groups with a higher reference speed; the identifiable nodes keep the same speed.

**Table 3:** Reference nodes at 2mps base speed.

Node	Speed	Run 1	Run 2	Run 3	Run 4	Average	Difference
A	0mps	0.04104	0.07174	0.07589	0.05473	0.06085	1%
B	2mps	0.05415	0.05641	0.05354	0.06097	0.056269	-7%
C	4mps	0.08870	0.07701	0.08356	0.08001	0.08232	36%
D	12mps	0.18471	0.19234	0.19043	0.20741	0.193723	220%
R. Nodes	2mps	0.05983	0.06167	0.05946	0.06082	0.060445	0%
ICC	0.977581						

**Table 4:** Reference nodes at 4mps base speed.

Node	Speed	Run 1	Run 2	Run 3	Run 4	Average	Difference
A	0m/s	0.08676	0.08171	0.07247	0.09574	0.084169	-19%
B	2m/s	0.08544	0.08578	0.08301	0.08596	0.085048	-18%
C	4m/s	0.11039	0.10037	0.11496	0.09974	0.106366	2%
D	12m/s	0.20439	0.19831	0.21068	0.21498	0.207091	99%
R. Nodes	4m/s	0.10426	0.10285	0.10307	0.10545	0.103906	0%
ICC	0.984365						

The result in Tables 3 and 4 are similar to Table 2; identifying nodes with higher speeds by a greater difference in LDED is possible, but struggling to identify slower nodes just by LDED.

## Conclusion

One unwanted consequence of mobility and high traffic is the loss of packets and link degradation. LDED takes into account the packets lost in a time window and creates a relative index to evaluate the quality of a node to connect to neighbors.

LDED focus on the links to surrounding neighbors, evaluating how trustworthy a node could be to pass packets to a near neighbor. Evaluating how trustworthy is a node is relative to the interaction with neighbors.

Each node evaluates LDED, assessing the links to the closest neighbors and calculates how much disconnection disorder is in the links between them. When all nodes move at the same speed the links between nodes have similar behavior and the average disconnection disorder is similar; meaning all nodes have the same consistency to communicate, not an LDED value of 0.

When a node is moving faster than the rest of nodes, the node will meet new neighbors and will miss links to others. The interaction will cause a high level of disconnection entropy disorder by the total connections made and lost.

A higher Link Disconnection Entropy Disorder means a node is passing in transmission range of a neighbor and shortly getting out, breaking contact from nodes. A node is unreliable if LDED is high or near 1.0.

Link breakage caused by channel saturation or other events have the same effect with less impact in LDED. Channel saturation makes the beacon mechanism to lose some packets and to take this as a link loss but recover in time with less impact to LDED than mobility.

LDED evaluate unreliable nodes and identify fast moving nodes without the need for specialized devices like GPS or sensors like infrared proximity. LDED takes simple count of packages in a timeframe to relate to near neighbors; every node is constantly evaluating and participating in LDED.

LDED uses the connection with neighbors as link, maintained via a beacon; in this work the metric is focused in network comunications; this metric could be used in other contexts by adapting the way neighbors are recognized and maintained. e.g. mobile agents are common tools to study dynamics in game models like the traveler’s dilema [57], fund strategy identification [58], and public goods game[59], with the objective of analyse cooperation, agglomeration and social instability. This traits are observable by the mobility of an agent. Chen et al. [60] investigates the cooperative behaviors among mobile agents in the public goods game, where the level of mobility measured by a metric such as LDED could denote the satisfaction level in past groups. When the agent has a high LDED, it would denote a rapid change of groups of neighbors, mostly by insatisfaction. If the metric is low, there is not much change in neighbors, indicating satisfaction within the visited groups.

## Supporting Information

S1 Data Simulation data results. Data from Omnet++ simulations used in results section.
(XLSX)
